# American Medical Association

**Published:** 1883-07-20

**Authors:** T. B. Greenley

**Affiliations:** Orel, Arkansas


### Southern Medical Record:

EDITORS:

T. S. Powell, M.D. W. T. Goldsmith, M.D. R. C. Word, M.D.

*R. C. WORD, M.D., Managing Editor.*

☞All Communications and Letters on Business connected with the Record must be addressed to the Managing Editor.

Vol. XIII. ATLANTA, GA., JULY 20, 1883. No. 7.

### ORIGINAL AND SELECTED ARTICLES.

### AMERICAN MEDICAL ASSOCIATION.

Editors Record:

Being present at the meeting of the American Medical Association, at Cleveland, I thought I would send you a condensed report of its proceedings, etc.

The Association convened on Tuesday morning, June 5th, with president John L. Atlee, M. D., in the chair. We were very eloquently welcomed to the hospitality of the Forest City, by General Myer.

The president's address laid no special claims to oratory, but in a very plain and succinct manner recited many incidents and peculiarities in the lives of the leading great doctors of his student days, and early life as a practitioner. He spoke of Physick Pancoast, Wistar, Horner, Hodge, Dorsey, Coxe, Chapman, Dewees and others whose names are household words with the profession. The remembrance of these men seemed to impress the speaker very pleasantly, and conveyed to the minds of the audience the fact that it was of life-long affectionate regard. The address, as a whole, was regarded not only as creditable to the author, but as of great interest.

After the transaction of some miscellaneous business, and the appointment by the various State delegations of a nominating committee, the Association adjourned to meet in sections in the afternoon.

In the section on Practical Medicine, etc., Dr. Hollister, of Illinois, being chairman, Dr. Meller, of Chicago, read a paper by Dr. Munay, of the Marine Hospital service, on Yellow Fever. The paper, in reference to treatment, advised immediate taking to bed, the administration of warm foot baths, and suggests that if hemorrhage in the stomach took place and should not be vomited, it should be eliminated by the bowels. It laid emphatic stress on the necessity of absolute quietude of both body and mind. The symptoms should be treated with the usual remedies, but great caution must be observed to avoid producing nausea.

Prof. Campbell, of your State, in discussing the paper, remarked that bleeding in some cases was beneficial, and cited examples; also advised free vomiting with hot water. Several other gentlemen of note participated in the discussion.

Dr. Wm. Morrow Beach, of Ohio, read a paper on Milk Sickness. Some discussion ensued, when the section was adjourned till next day.

In the section on Obstetrics, etc., was read a paper by Dr. Byford, of Illinois, on "Chronic Intero-pelvic Inflammation." The Doctor endeavored to show that para and peri metritis does not constitute the entire character of inflammations of the pelvis; and spoke extenso of the necessity of being very careful in the examination of the pelvic organs when inflamed, and when much tenderness existed advised the use of anæsthetics. He laid down several precautionary rules by which we should be governed in such examinations and operations.

The next paper was on "Post-Partum Polypoid Tumors," by Dr. Landis, of Ohio. He spoke of four forms of this disease, and described two cases in detail.

The third paper was on "The Restoration of the Perineum, by a New Method," by Dr. Marcy, of Massachusetts. He uses in his method of restoration German silver wire, which is electric, and on this account, in his estimation, preferable to other sutures.

The next paper was on "Enterotomy as a Complication in Ovariotomy or Oophorectomy," by Dr. Sutton, of Pittsburg. He related two cases of removal of several inches of small intestine—one by Prof. Billroth, and the other by himself. Both cases recovered. His case was a lady, from whom he removed four inches of intestine a few months ago, and was the first successful operation of the kind performed in this country.

In the section on State Medicine, Dr. Gihon read quite a lengthy paper, in which he depicted great deficiencies in the education of many of our professional brethren. He illustrated his position by citing many instances of ignorance, both in medical knowledge and primary education, some of which were quite amusing as well as mortifying to our feelings; especially the latter, when we think of their being graduates of our most ancient and renowned institutions. The doctor became familiar with this fearful amount of ignorance while he was a member of the Examining Board of the United States Navy. He gave in detail the answers of some twenty-five applicants for surgeonships in the navy, most of whom were graduates of the oldest and considered the best medical schools in the country. I will only give you a few samples of the learned answers of these M. D. applicants: One said pneumothorax meant a collection of pus in the chest; another, that the boiling point of Fahrenheit was 300°; that centigrade not as high, and Reaumer higher, and could not tell what the zero of Fahrenheit was without having the others to compare by; and that the normal temperature of the body was 112° to 140°. A third could not recollect the country nor language of Hyppocrates, but that Galen lived before him, and that Virgil died in the 15th century. A graduate from this same school, during his attendance to be examined, was taken sick, and on his return excused his absence by saying he had been sick with "cholera *infantum*." Another graduate from a New England School said "the crepitant rhoncus occurs whenever a membrane becomes dry; that Plymouth was set tled by Columbus, who gave the town its name; and that each State and Territory has one Senator. Another graduate of a celebrated New York school, said that Galen introduced vaccination, or inoculation in the 17th century. Another New York M. D. regarded bronchotomy as a practical operation, and described it.

The doctor also gave us some samples of orthography gathered from the manuscripts of some of the candidates for examination. He stated that not more than 32 per cent. of those who applied for service in the navy passed examination, although the most of them came highly recommended, both as it regarded their medical acquirements and social standing.

Dr. Gihon seemed to think that the onus of the charge of ignorance and incapacity, to a great extent, depended on the want of action on the part of the American Medical Association, and suggested that it was time that this body was taking steps to correct such a lamentable condition of things. He seemed to entertain the opinion that we might as well fellowship the homeopath, as some of our own brethren who are so benightedly ignorant. He calls loudly on the Association to use its great influence to elevate, not only the standard of graduation, but also that of collegiate acquirements. With these ends in view he introduced some resolutions in the section on State Medicine, which were discussed by several gentlemen.

The section on Surgery, etc., was well attended, with Dr. Peck, of Iowa, in the chair.

Dr. Vance, of Ohio, read the first paper, entitled, "Radical Cure of Hernia, by a New Method." He had performed this operation nineteen times with satisfactory results. His plan applies to oblique inguinal hernia. His plan is to revivify and bring together the lips of the opening by means of a deep-seated suture passed subcutaneously with a semi-circular needle. By this means the previous wide open hernial canal becomes a closed valve, which resists the reprotrusion ot the abdominal viscera. The paper was well received.

Dr. Allen, of Cleveland, read a paper entitled, "Comparison of Antiseptic and non-Antiseptic Methods of Treatment." The conclusions of the doctor were that antiseptics were least useful in abdominal surgery; that the spray is the least important of all the details in antiseptics; that it might be most beneficial in the opening of joints, especially in hospitals; that, although danger may result from poisonous effects, yet these dangers are diminishing from a better knowledge of their use. The doctor summed up by saying that he believed the use of antiseptics in surgery afforded better results than any method at present in use.

Dr. Martin, of Massachusetts, seemed to think that Listerism would soon be a thing of the past, which was firmly denied by Dr. Nankin, of Pennsylvania. The paper provoked much discussion.

The next paper was read by the venerable Dr. Gross, on "The Value of Early and Late Operations in Morbid Growths, Especially Malignant." The doctor looks to be in excellent health, and was greeted with loud applause. The paper, of course, was very able, and portrayed the great advantages of early operations in all morbid growths, and especially those of a malignant character. His reasons for early operations were thus stated: "The less risk of shock and of hemorrhage; the more effectual riddance of diseased structure; the diminished probability of septicemia or blood poisoning; the avoidance of unsightly sores; and the less risk of a recurrence of the morbid action, either at the seat of the operation or in other parts of the body." Of course the paper was well received, and the principles it taught regarded as a law unto us all.

Dr. Henry Martin, of Massachusetts, followed in a paper on the

"Treatment of Synovial Diseases by a New Method," in which he advocated the drawing off of the fluid by aspiration and the application of the rubber bandage. He had treated a great number of cases successfully by this method.

Dr. Holt, of Massachusetts, and Dr. Neuman, of New York, also read papers, both of interest to the profession.

In the section on Diseases of Children, Dr. Earle, of Chicago, read a paper on "Cephalæmatoma in the Newborn." This is regarded as quite a rare disease. It consists of a painless, elastic, soft, fluctuating tumor, situated on one of the parietal bones. The doctor had seen four cases in a long practice. He thought, as a rule, it required but little treatment. There were other papers for this meeting, but their authors not having arrived their reading was postponed.

In the section on Dental and Oral Surgery, Dr. Marshall, of Chicago, read a paper on "Denudation or Erosion of the Teeth." The doctor described this disease as beginning with the enamel, and gradually involving the subjacent dentine, without any of the appearances or characteristics of caries. It consists of a general wasting of the enamel and dentine. The paper was regarded as quite able, and was discussed by several gentlemen. About the close of this session of the section, three gentlemen entered the room and objected to Dr. Goodwillie presiding as chairman of the section. The doctor said he was not acquainted with the gentlemen, and did not know whether they were entitled to speak. The gentlemen were Drs. Noyes, Nichol and Hinton, of New York. They contended that Dr. G. registered under protest of the judicial council. It is said he favors consultation with the homeopaths. Whether this charge is true or not the doctor resigned his office as chairman, when Dr. Williams was elected.

Several papers were read before the section on Ophthalmology, etc. Dr. Turnbull, of Pennsylvania, read "Paralysis of the Facial Nerve in connection with Diseases of the Ear." Dr. Harper, of Illinois, "Hysterical Amblyopia." Dr. Jarvis, of New York, "Tonsilotomy by Ecrasement." Dr. Carl Seiler, of Pennsylvania, "Nitrate of Silver on the Mucous Membrane of the Throat." Also several other papers of interest by other gentlemen, were read.

When I sat down to give you a short account of the transactions of the Association, I thought I would mention most of the papers read as well as a synopsis of the business matters, but I find it would make an article entirely too lengthy for your Journal, and therefore will only allude to what took place after the first day.

It was resolved by the Association to publish a weekly journal instead of the annual volume of transactions. It strikes us that this will be a much more eligible form of placing the valuable papers before the professional world than in a single volume published nearly a year after each meeting. Dr. N. S. Davis, of Chicago, was unanimously elected editor-in-chief, and the Journal will be published at Chicago. Each member is entitled to a copy by paying his annual dues; and every doctor outside of the Association can obtain a copy for five dollars a year. It will be about the size of the Philadelphia Medical News.

The reports of the chairmen of the various sections were generally very able, and some of them not only able but quite eloquent—notably, that of Dr. Hollister, of the practice of medicine. I will not have space in this letter even to name the papers read on the 2d, 3d and 4th days of the meeting, but suffice it to say that they were admitted to be able; and take the proceedings altogether, will compare in interest and ability to any for several years.

There were nearly 1,100 members registered, besides many visitors. The next meeting will be at Washington City.

Dr. Austin Flint, Sr., was elected president. This compliment to the doctor was considered by the Association as due him on account of the great eminence he has attained in the profession as a writer and hard worker generally; and like the late president, Atlee, is advancing in years.

I have no doubt but that the next president will be from the South, and am inclined to think it will be Prof. Campbell of your State. He stands high as an able and efficient member of the Association.

Upon the whole, we had quite a pleasant, interesting and able meeting, and one, I hope, that will prove beneficial to our guild.

With much respect, etc.,

T. B. GREENLEY, M. D.

*Orel, Arkansas.*

